# Incision of the Urethra

**Published:** 1848-12

**Authors:** 


*Incision of the Urethra.*—We mentioned, in the report of the last meeting of the Academy, the conclusion of a paper on this subject, by M. Civiale. We have noticed another instance in which the division of the urethra was an operation of great utility: we refer to ancient fistula of the passage. In several cases of abnormal apertures existing in the canal,—the consequence of gangrenous inflammation,—M. Segalas first proposed, and Dr. Ricord performed it. In one patient, the fistular opening, situated at the union of the penis with the scrotum, was nearly one inch in length, and had lasted twenty years. The object of opening the urethra in the perinæum, was to permit the escape of urine from the incision, through which a bougie was introduced into the bladder, thus allowing the surgeon to employ the autoplastic method for the closure of the ancient fistula, the cicatrisation of which would be rendered most difficult by the contact of the wound with urine. The fresh incision performed in the perinæum healed without difficulty, and the infirmity was completely removed. The invention of this method, crowned with success in several instances, was recompensed by a prize awarded by the Institute in the year 1842 to Drs. Segalas and Ricord.—*Ibid, from Gaz. Med.*

